# Supplementary material for: Methodological proposal to identify the nationality of Twitter users through random-forests
Source: PLoS One. 2023 Jan 31;18(1):e0277858. doi: 10.1371/journal.pone.0277858 (PMC9888676; doi:10.1371/journal.pone.0277858)
Supplement: Attachment — Submitted filename: Response to Reviewers.docx [file pone.0277858.s001.docx]

Response to Reviewers.

We thank you for your observations.

**Observation 1**. *Ensure that your manuscript meets PLOS ONE's style requirements, including those for file naming*.

The following changes were made to the manuscript:

The authors section was completely modified, it was adjusted to what is indicated in formatting sample title authors

Keywords were removed

Increase space between Abstrac and first line

Increase space between Related Work and the first line

Fig**.** 4. to Fig 4. : adjusts to what is indicated in format sample main body

Fig**.** 5. to Fig 5. : :adjusts to what is indicated in format sample main body

***Fig 7****. Extract from resultsNI table , Nicaragua cas*e it is changed to ***Fig 8****. Extract from resultsNI table , Nicaragua case.*

The figure is the same, the error was the number.

**Observation 2**. *We note that the grant information you provided in the ‘Funding Information’ and ‘Financial Disclosure’ sections do not match.*

You are right. We are wrong. We are confused, we have not received financial support in our research. However, we are confident that the Specialized University of the Americas and the University of La Rioja will support us in paying the costs of publishing the article. However, we have our own resources to pay.

In the ‘Funding Information’ section.We will write that we have not received funds for research.

**Observation 3. About financial disclosure.**

As explained in observation 2, we were wrong. Contributions of $902.50, $902.50 are expected, and it is to pay the publication costs in case the article is approved, it is not research funding. We have requested this support from the universities, but it is not certain. We have our resources.

"The funders had no role in study design, data collection and analysis, decision to publish, or preparation of the manuscript." .

**Observation 4**. *About Data Availability statement, you have not specified.*

The study data mainly used 30 tables stored in two repositories:

Repository 1 has 12 tables:

https://github.com/damian-quijano/identify_nationality_twitter

http://dx.doi.org/10.5281/zenodo.7254532

Repository 2 has 18 tables:

https://github.com/damian-quijano/-identify_nationality_twitter2

http://dx.doi.org/10.5281/zenodo.7254534

This information was included in the submit(first time) in the section Additional information- Data Availability

It is fully accessible. All the tables and data mentioned in the manuscript are downloadable from the repositories.

This information, about full access to the data, is mentioned in section *2.2 Data collection* of the manuscript.
